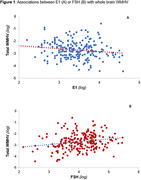# Sex hormones and white matter hyperintensities in midlife women in the MsBrain study

**DOI:** 10.1002/alz.092810

**Published:** 2025-01-03

**Authors:** Rebecca C Thurston, Minjie Wu, YueFang Chang, Howard J Aizenstein, Carol A. Derby, Pauline M Maki

**Affiliations:** ^1^ University of Pittsburgh, Pittsburgh, PA USA; ^2^ Department of Bioengineering, University of Pittsburgh, Pittsburgh, PA USA; ^3^ Department of Neurology, and Department of Epidemiology and Population Health, Albert Einstein College of Medicine, Bronx, NY USA; ^4^ Department of Psychiatry, University of Illinois at Chicago, Chicago, IL USA

## Abstract

**Background:**

Sex hormones are frequently implicated in the development of cerebral small vessel disease among midlife women. However, few studies directly measure endogenous sex hormones and consider them in relation to white matter hyperintensities (WMH), indicators of cerebral small vessel disease. Further, existing work on hormones, menopause, and the brain typically focuses on ovarian estradiol (E2), with limited consideration of estrone (E1), the primary postmenopausal estrogen, or follicle stimulating hormone (FSH), an indicator of ovarian age. We tested the associations of E2, E1, and FSH in relation to WMH volume (WMHV) among late midlife women. We considered both whole brain WMHV and the spatial distribution of WMHV.

**Methods:**

222 women ages 45‐67 (mean = 59.29 years, 99% postmenopausal), not taking hormone therapy, and free of a history of cardiovascular disease were recruited for the MsBrain study. Procedures included physical measures; phlebotomy for sex hormones (E1, E2, FSH; estrogens assessed via LC‐MS/MS), glucose, insulin, and lipids; and 3T magnetic resonance imaging. Associations of E1, E2, and FSH with WMHV were tested in linear regression models with covariates age, race/ethnicity, education, and in a separate step, adjusted for cardiovascular disease (CVD) risk factors [body mass index, smoking, blood pressure level and medications, insulin resistance, lipids]. Additional models considered regional WMHV (deep, periventricular, frontal, temporal, parietal, occipital).

**Results:**

In age, race, and education‐adjusted models, lower E2 [B(SE) = ‐.12(0.05), p = .02], lower E1 [B(SE) = ‐0.26(0.10), p = 0.007], and higher FSH [B(SE) = 0.26(0.07, p = 0.0005] were each associated with greater whole brain WMHV. When further controlling for CVD risk factors, associations of E1 [B(SE) = ‐.21(.10), p = .04] and FSH [B(SE) = .18(.09), p = .04] (but not E2) to whole brain WMHV remained (Figure 1). Considering the spatial distribution of WMHV, lower E1 was associated with more frontal, deep, and temporal WMHV; and higher FSH with more frontal and periventricular WMHV.

**Conclusions:**

Findings indicate the importance of endogenous sex hormones to late midlife women’s cerebrovascular health. They underscore the value, when investigating aging women’s brain health, of moving beyond a sole focus on E2 to also consider E1 and FSH, hormones particularly relevant in the postmenopause.